# Study on the canopy structure and light distribution of *Hippophae rhamnoides* at different ages

**DOI:** 10.3389/fpls.2026.1830572

**Published:** 2026-05-28

**Authors:** Kaiwen Tan, Yawen Wang, Guozhen Yan, Liwen Zhang, Jin Lei, Junyang Wang, Yan Wang, Xiaoliang Dong, Cheng Tang, Qiang Jin

**Affiliations:** 1 College of Agriculture, Shihezi University, Shihezi, China; 2 College of Life Science, Shihezi University, Shihezi, China; 3 College of Urban and Environmental Sciences, Shihezi University, Shihezi, China; 4 Urumqi Forestry and Grassland Bureau, Urumqi, China; 5 College of Mechanical and Electrical Engineering, Shihezi University, Shihezi, China; 6 Xinjiang Silk Road Sea Buckthorn Technology Co, Tacheng, China; 7 College of Horticulture and Forestry, Tarim University, Alar, China

**Keywords:** gap fraction, random forest, redundancy analysis, stand age, structural equation modeling

## Abstract

The canopy structure of seabuckthorn has a profound effect on its light environment and ecological function with the dynamic evolution of stand age. To quantitatively analyze the driving mechanism of stand age and canopy structure on light distribution, PAR and key canopy structure parameters (LAI, PALe, OmegaE and Gap Fraction) were measured simultaneously along the vertical profile (bottom, middle and top) of 3-, 4-, 8- and 11-year-old seabuckthorn plantations by using TRAC. Two-way analysis of variance, structural equation modeling (SEM), redundancy analysis (RDA) and a variety of machine learning models were used for analysis. The results revealed that stand age and relative height had significant effects on all canopy parameters and PAR (p< 0.001). SEM revealed that the unit Gap Fraction had the strongest direct positive effect on PAR (b = 0.826), whereas the LAI indirectly affected PAR mainly by negatively regulating the unit Gap Fraction (b = −0.628). RDA revealed that stand age was the dominant factor driving the variation in canopy structure (LAI, PALe), with an independent inter-pretation rate of 31.72%. The random forest model performed best in the classification task of stand age inversion on the basis of canopy characteristics (accuracy rate of 85.19%). This study provides an important theoretical basis and quantitative tool for optimizing light distribution, coordinating growth and fruit-ing, and realizing the coordinated improvement of ecological and economic benefits through canopy structure management (such as reasonable pruning to maintain an appropriate Gap Fraction) of seabuckthorn economic plantations.

## 1 Introduction

Seabuckthorn (*Hippophae rhamnoides* L.), a pioneer tree species with strong vitality, has long played a key role in the ecological development of China. It has been widely used to create soil and water conservation forests and windbreak sand-fixing forests and has made great contributions to the restoration and management of fragile ecological areas (Liu et al., 2020). With increasing understanding of the multifunctionality of seabuckthorn resources, their value is far greater than that of an ecological barrier. Owing to its unique growth habit of drought tolerance and barrenness tolerance, as well as the extremely high nutritional and medicinal value of the fruit, seabuckthorn has transformed from a simple ecological guardian to an ‘ecological economic forest’ with integrated ecological and economic benefits (Olas, 2018). Exploring the development mode, potential and challenges of seabuckthorn in the construction of ecologically economic plantations is of great theoretical and practical importance.This study aims to provide directly applicable quantitative data and conclusions for the development of seabuckthorn economic plantations through a quantitative investigation of canopy structure and light distribution, this study can also partially fill the gaps in research on the canopy structure and light distribution of seabuckthorn plantations.

Recent advances in Hippophae rhamnoides research span from bioactive compounds to genomic resources. Jaśniewska and Diowksz (2021) reviewed the wide spectrum of bioactive compounds in seabuckthorn and proposed its application as a functional additive fordeveloping novel food products to prevent lifestyle diseases (Jaśniewska and Diowksz, 2021). Yang et al. (2024) presented a chromosome−level genome assembly of Fructus hippophae (a hybrid variety of seabuckthorn with H. rhamnoides ssp. mongolica as the female parent and H. rhamnoides ssp. sinensis as the male parent), achieving a genome size of 918.59 Mb anchored onto 12 pseudochromosomes, and predicted 36,475 protein−coding genes with a BUSCO completeness of 98.80%; this high−quality genomic resource provides a foundation for pan−genomic studies and for elucidating the mechanism of sex differentiation in sea buckthorn (Yang et al., 2024). Melnikova et al. (2025) performed whole-genome sequencing on 55 Russian-bred seabuckthorn varieties with different fruit characteristics and lineages, identified DNA polymorphisms in genic regions, and revealed genetically distinct groups corresponding to genotype lineages; these genomic data provide a foundation for QTL/QTN mapping and marker-assisted selection, therebysupporting both basic and applied research on sea buckthorn genetic diversity and breeding (Melnikova et al., 2025). Yan et al. (2026) integrated stage-resolved transcriptomic profiling with co−expression network analysis to characterize the regulatory landscape of axillary bud activation and branch elongation in the woody perennial Hippophae rhamnoides, revealing temporally coordinated auxin−responsive modules rather than bulk hormonal accumulation alone (Yan et al., 2026). However, these studies primarilyfocus on molecular genetics, active compounds, or organ development, while systematic quantitative research on age-driven canopy structural heterogeneity and its effects on light distribution remains limited.

Canopy structure serves as the critical bridge connecting genetic traits to ecological functions, directly determining the spatiotemporal pattern of photosynthetically active radiation (PAR), and consequently affecting growth, fruiting, and the synergistic improvement of ecological and economic benefits. Therefore, elucidating the coordinated evolution of canopy structure and light environment under different stand ages is essential for optimizing management strategies in seabuckthorn economic plantations.

With increasing seabuckthorn stand age, the internal structure of the canopy will undergo a series of complex and significant evolution processes (Tun et al., 2018; Zhuk et al., 2015). These structural changes not only have a profound impact on the light environment in the forest and subsequently affect the growth of trees but also have a chain reaction to the function of the entire ecosystem, thus affecting the stability and productivity of the ecosystem at multiple levels (Hooper et al., 2005). PAR is the direct energy source that drives the photosynthesis of trees, and its spatial and temporal distribution characteristics in the canopy are the result of the dynamic interaction between canopy structure and stand age (Duan et al., 2025). With increasing stand age, the morphological structure of trees and the level and density of the canopy change accordingly.

Zhang et al. (2021) proposed a method for estimating canopy-captured PAR (fPAR) based on a three-dimensional model of the canopy measured via aerial photography. In this study, three experimental almond orchards in California, including the main variety ‘Nonpareil’, were tested. Through the development of a complete data collection and processing process (virtual orchard, VO), the characteristics of canopy coverage and canopy volume index were extracted and compared with the fPAR measured by the traditional mobile light stripe platform. The results revealed that the fPAR estimated via VO was strongly correlated with the actual almond yield (*R*^2^ = 0.96) (Zhang et al., 2021). Wang et al. (2023) analyzed the effects of different canopy positions and harvest times on the severity and quality of Fuji apple fruit watercore disease. The severity of fruit watercore disease increased with increasing harvest time, and the upper and west sides of the canopy were more severe, which was closely related to the light conditions and carbohydrate assimilation supply (Wang et al., 2023). Yan et al. (2025) analyzed the effects of different pruning methods on the canopy structure, light distribution and fruit spatial distribution of Korla fragrant pear and reported that canopy size and light distribution were the key factors affecting fruit distribution (Yan et al., 2025). Anthony and Minas (2025) evaluated the effects of different pruning systems on the canopy structure, light distribution and physiological characteristics of peach trees. Different pruning systems significantly affect the light distribution and growth potential diffusion of the canopy, which in turn affects the yield and quality of the fruit (Anthony and Minas, 2025). Studying the light distribution of the tree canopy is highly important for determining the yield increase of fruit trees and the health of trees (Zhang et al., 2025). At present, there is still a lack of systematic quantitative research on how the canopy structure and light environment of shrub forests with important ecological functions, such as seabuckthorn, evolve regularly with stand age and what the key driving paths are.

In this study, 3-year-old, 4-year-old, 8-year-old and 11-year-old seabuckthorn plantation were selected to form a stand age sequence of spatial generation time, 3- and 4-year-old stands represent the vigorous vegetative growth stage, characterized by active canopy construction; the 8-year-old stands represent the transition point to full fruit-bearing, with intensified competition between vegetative and reproductive growth; and the 11-year-old stands represent the mature stage with a gradually closing canopy (Zhao et al., 2010). This age sequence allows a systematic capture of the dynamics of canopy structure and light environment evolution throughout the transition from vegetative-to-reproductive dominance in seabuckthorn plantations, with the aim of quantifying the main effects and interaction effects of stand age and canopy relative height on the PAR and key canopy structure parameters. The relationships between canopy structure parameters (LAI, PALe, OmegaE, and Gap Fraction) and PAR and PPFD were analyzed. The main direction and key driving factors of light environment variation in the seabuckthorn canopy at different stand ages were identified. Through this research, specific parameters can be provided for the quantitative characteristics of seabuckthorn plantations (e.g., OmegaE, Gap Fraction), as well as the relative contributions of stand age and relative height to light distribution. These currently missing parameters are crucial for designing science−based pruning and density management strategies for this important economic tree species. In this study, conventional models (ANOVA, SEM, RDA) are used to explore causal mechanisms, while machine learning models are employed as a practical tool to invert stand developmental stage from easily measurable canopy characteristics, which has direct and significant value for the management of seabuckthorn plantations.

## 2 Materials and methods

### 2.1 Selection of the study area

**Figure 1:**
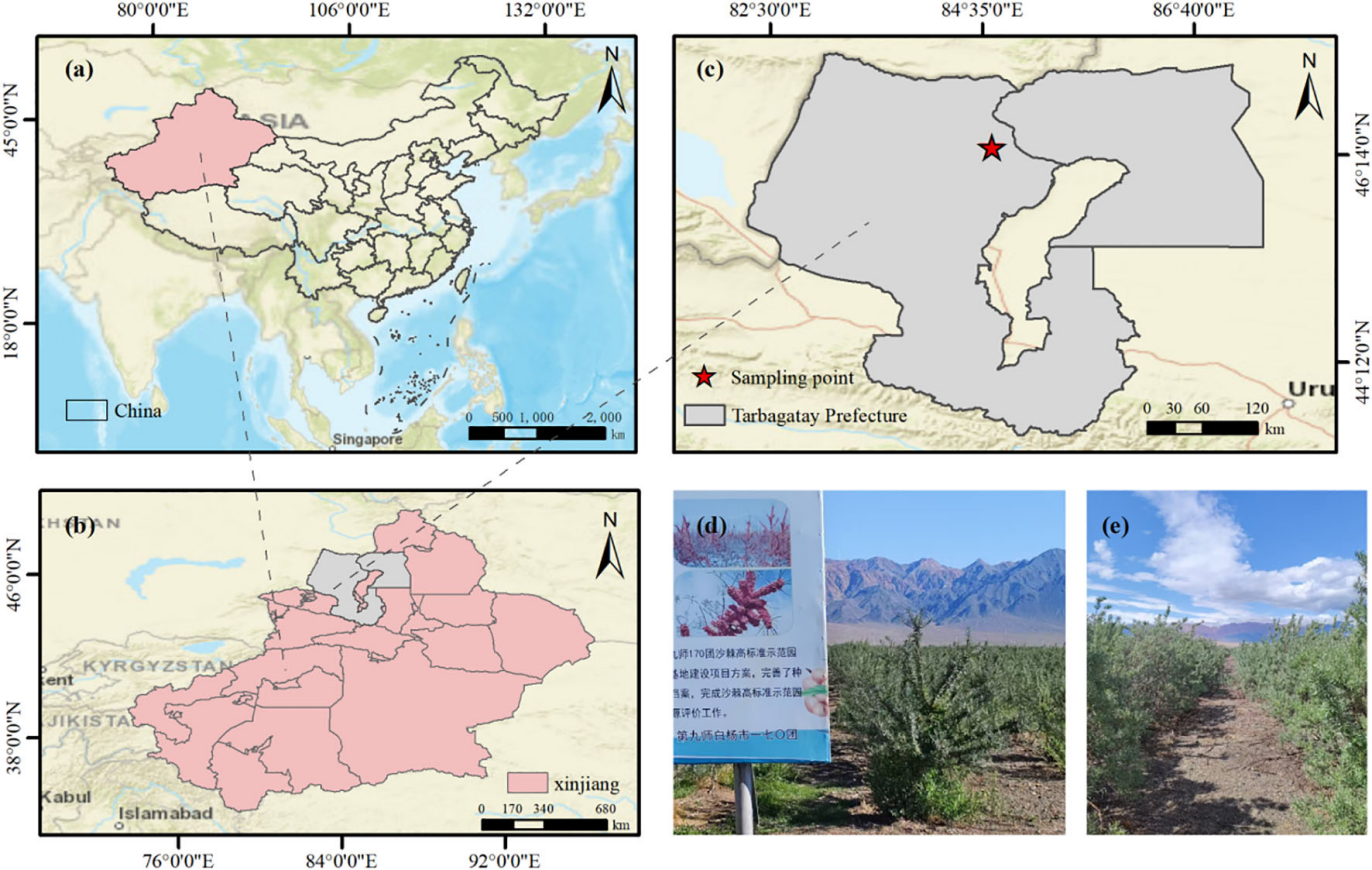
The specific location of the sampling site. **(a)** the country where the experimental site is located; **(b)** the province where the experimental site is located; **(c)** the prefecture/region within the province where the experimental site is located; **(d)** and **(e)** specific scene conditions of the experimental site.

**Figure 2:**
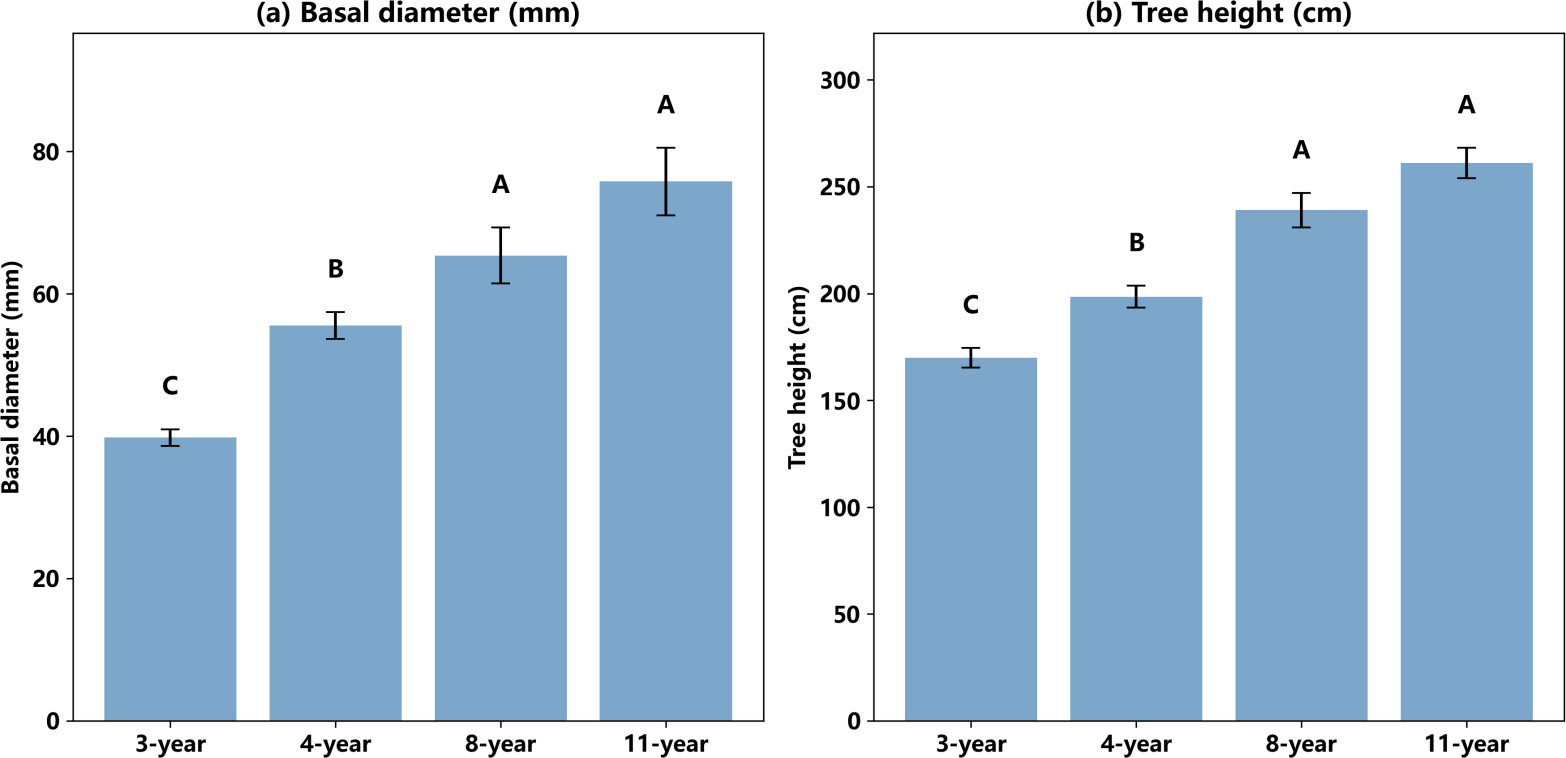
Differences in stand characteristic. **(a)** Basal diameter (mm) of seabuckthorn trees across different stand ages groups (3, 4, 8, and 11 years); **(b)** Tree height (cm) across different stand ages groups. Different lowercase letters (a, b, c) indicate significant differences among stand ages groups at p < 0.05. Error bars represent standard deviation.

The study was carried out on a seabuckthorn plantation in 170 regions (84°38’20”E, 46°17’40”N) in Emin County (**Figure 1**), the ninth division of Tacheng Prefecture, Xinjiang Uygur Autonomous Region, China. The experimental area has a typical temperate continental desert climate in China. The whole year is characterized by drought and little rain, high evaporation, sufficient light, an abundant heat source and a large temperature difference between day and night. The area is mainly distributed in foxal soil, sierozem, brown calcic soil, meadow soil and gravel soil, which are highly adaptable to seabuckthorn and other crops. To construct the stand age sequence, 3-year-old, 4-year-old, 8-year-old and 11-year-old seabuckthorn plantation with basically the same site conditions were selected as the research objects, The differences in stand factors (tree height, basal diameter) among different stand ages are shown in the figure (**Figure 2**) (Guo et al., 2024). Standard plots were set up at each stand age, and 30 sample trees were randomly selected from 3-year-old, 4-year-old, 8-year-old and 11-year-old seabuckthorn plantation for investigation (Yue et al., 2018). The sample selection is shown in **Table 1**.

**Table 1:** Sample selection number of strains.

Stand age	Selection sample number
3-year-old	30
4-year-old	30
8-year-old	30
11-year-old	30

### 2.2 TRAC

**Figure 3:**
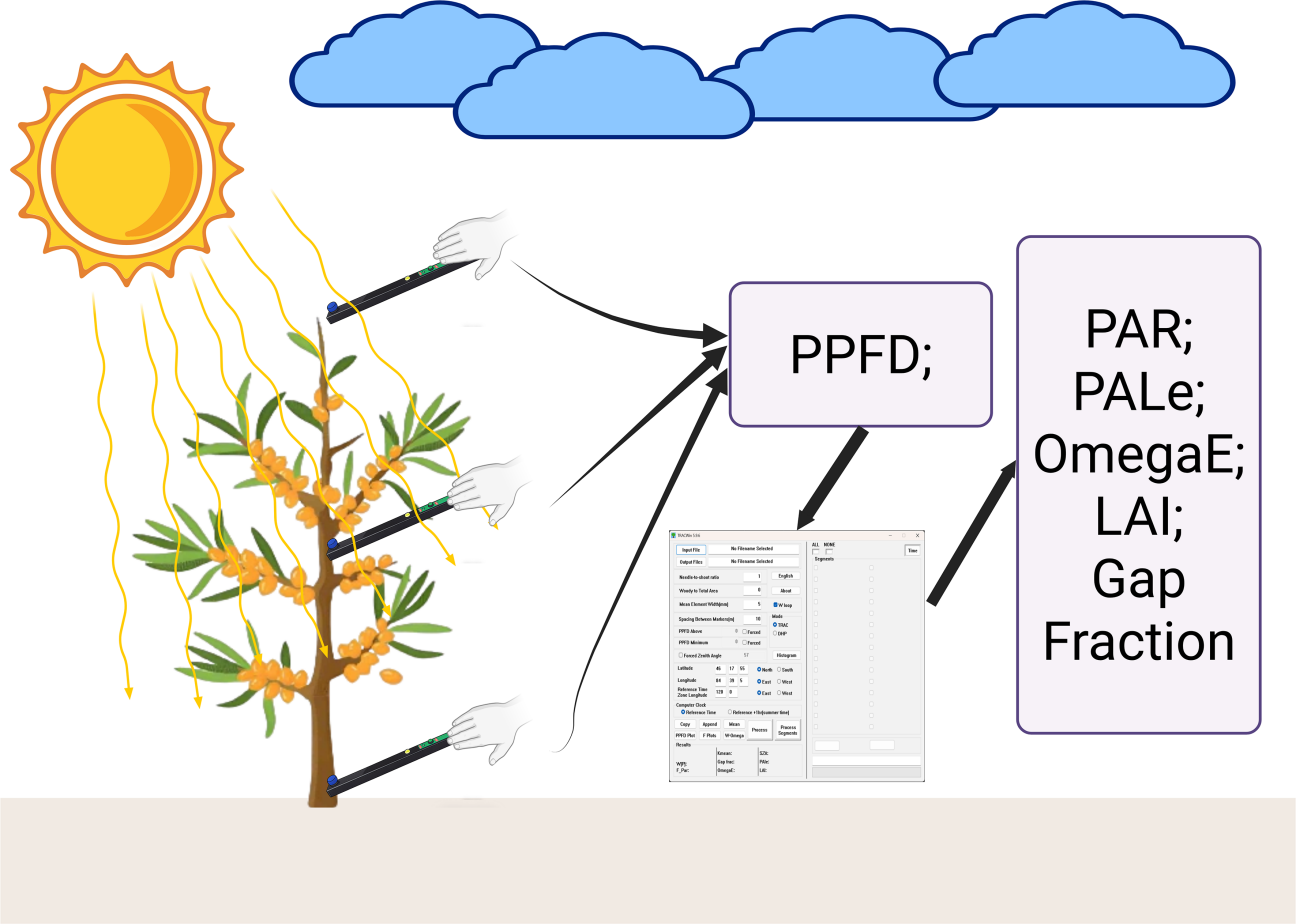
TRAC operation method.

TRAC (Tracing Radiation and Architecture of Canopies) canopy analyzer is a portable optical instrument specifically designed to quantitatively assess the structural characteristics of plant canopies and their capacity to intercept photosynthetically active radiation (PAR). Unlike traditional instruments that rely solely on gap fraction, TRAC records the photosynthetic photon flux density (PPFD) of direct solar radiation penetrating the canopy at a high sampling frequency (30 Hz), thereby obtaining gap size distribution information and correcting the underestimation of leaf area index (LAI) caused by nonrandom leaf distribution (clumping effect) (Chen and Cihlar, 1995). The TRAC main unit is equipped with a high-sensitivity PAR sensor (measurement band 400–700 nm). During field measurements, the operator holds the instrument horizontally and walks at a constant speed of approximately 0.3 m·s^−1^. The sampling location must be under the canopy of trees that provide shade, i.e., where shade is present (**Figure 3**). The sensor records the relative PPFD values of transmitted radiation in real time. The collected data are transmitted via Bluetooth to an Android data logger with a dedicated app, and subsequently imported into the TRACwin software for post-processing (Chen et al., 1997). The calculation procedures for each parameter are described below (**Table 2**).

**Table 2:** Measurement indicators and calculation formulas.

Measure index	Index explanation	Formula	Formula number
PPFD	Photon flux density (μmol·m^−2^·s^−1^)	${\text{PPFD}}_{\text{abs}}=\frac{{\text{PPFD}}_{\text{raw}}}{4096}\times 2500$	(1)
PAR	Photosynthetically active radiation (μmol·m^−2^·s^−1^)	${\text{PAR}}_{\text{abs}}={\text{PPFD}}_{\text{abs}}$	(2)
PALe	Effective plant area coefficient	$\text{PALe}=\frac{\text{LAI}}{1-\alpha }\;(\text{typically }\alpha =0)$	(3)
OmegaE	Clumping index	${\text{Ω}}_{E}=\frac{\int_{0}^{\infty }(1-F_{m}\left(x\right))dx}{\int_{0}^{\infty }(1-F_{r}\left(x\right))dx}$	(4)
LAI	Leaf area index	$\text{LAI}=-\frac{\cos\;\theta }{G\left(\theta \right)}\cdot \frac{\ln\;(F_{mr})}{\Omega_{E}}$	(5)
Gap Fraction	Unit clearance area	$F_{m}=\frac{\text{Total gap length}}{\text{Total transect length}}$	(6)

Variables are defined as follows (based on TRAC manual),.

• PPFD_raw_, raw relative PPFD value (dimensionless) — TRAC records values from 0 to 4095.

• PPFD_abs_, PAR_abs_, absolute photon flux density (*µ*mol·m^−2^·s^−1^).

• LAI, leaf area index (m^2^·m^−2^).

• *α*, leaf absorption coefficient (dimensionless, typically 0 for green leaves).

• Ω*_E_*, clumping index (dimensionless), quantifying foliage grouping.

• *F_m_*(*x*), *F_r_*(*x*), gap fraction measured in actual canopy and in random canopy, respectively.

• *θ*, solar zenith angle (degrees or radians).

• *G*(*θ*), projection coefficient of leaf area in direction *θ* (commonly 0.5 at *θ* = 57.3^°^).

• *F_mr_*, ratio of measured gap fraction to random gap fraction (*F_m_/F_r_*).

• *F_m_*, measured gap fraction, i.e., proportion of gaps along the transect (dimensionless).

• *Total gap length*, *Total transect length*: total length of gaps and total length of the measurement transect (same unit).

### 2.3 Data collection

At the peak of the growing season (July 6 to July 18), the following parameters were measured simultaneously at the relative tree height (0: bottom of the canopy; 0.5: middle of the canopy; 1: top of the canopy) via a plant canopy analyzer (TRAC) along the vertical section of the canopy: PPFD, PAR, PALe, OmegaE, LAI and Gap Fraction (**Table 2**; **Equations 1**–**6**) (Chenu et al., 2008). In this study, relative height was used as a variable factor instead of absolute height. Since stand age itself is highly correlated with absolute height, the dimensionless “relative height” was adopted in order to focus on the effect of vertical position within the canopy on a unified scale, thereby enabling standardized comparisons across stand ages. Finally, a total of 214 valid samples were obtained. When the relative height was 1, there was no crown above the TRAC sensor, so it was regarded as the CK. The measurement time period was selected as 9:00–11:00 in the morning and 4:00–6:00 in the afternoon. The weather was selected as sunny weather, and the measurement was better when this time period and weather conditions were selected (Fang et al., 2019). Since the influence of the sun’s position is small when TRAC is used, the solar zenith angle (SZA) is omitted from the variable.

### 2.4 Statistical analysis methods and models

In this study, two-way ANOVA was first performed on the obtained sample data, and ‘stand age’ and ‘relative height’were used as fixed factors to analyze their independent and interactive effects on each variable (Gonzalez, 2009). A correlation analysis of these variables was subsequently performed, and SEM was constructed to construct a prior model. Since there was a linear relationship between the PPFD and PAR, the PPFD was discarded to quantify the causal relationships among the PALe, OmegaE, LAI, Gap Fraction and PAR (Doncaster, 2007). Then, RDA was carried out, with stand age, relative height, PALe, OmegaE, LAI, and Gap Fraction as explanatory variables and PAR as the response variable. RDA was carried out to quantify theexplanatory power of environmental factors and to explain the influences of stand age, canopy height and canopy structure on PAR under the canopy (Legendre and Legendre, 2012). Five models, the KNN, SVM, Random Forests, CNN, and CNN-LSTM models, were used toclassify the samples according to the stand age (Masood et al., 2023; Abohashish et al., 2025).

## 3 Results

### 3.1 Regulation of canopy structure parameters and light distribution by stand age and relative height

**Figure 4:**
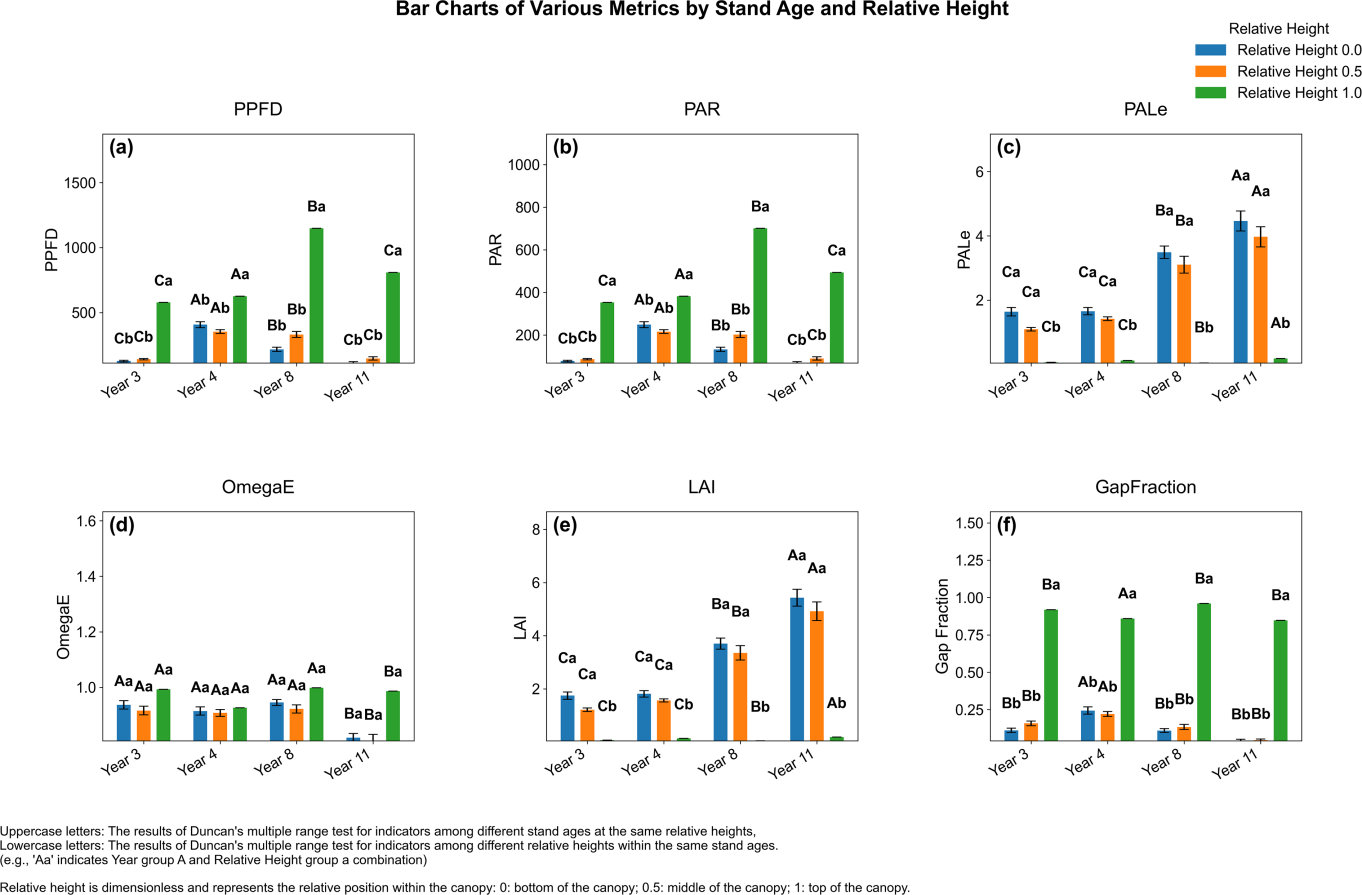
Histogram of each index under different stand ages and relative heights. **(a)** shows the changes in Photosynthetic Photon Flux Density (PPFD) under different stand ages and relative heights , indicating that PPFD is highest at the canopy top and decreases significantly in the middle and bottom of the canopy with increasing stand age; **(b)** shows the vertical distribution pattern of Photosynthetically Active Radiation (PAR), reflecting how canopy light interception capacity changes with stand age and vertical position; **(c)** shows the variation of the effective plant area coefficient (PALe) with stand age and relative height, indicating that increasing stand age significantly enhances the effective light interception area of the canopy; **(d)** shows the distribution of the clumping index (OmegaE) across different stand ages and relative heights, revealing the spatial arrangement characteristics of seabuckthorn leaves and their effect on light scattering; **(e)** shows the variation of leaf area index (LAI) with stand age and relative height, reflecting the dynamic evolution of canopy structural complexity as stand age increases; **(f)** shows the differences in gap fraction across different stand ages and relative heights, indicating that canopy closure increases with stand age and that the gap fraction at the canopy bottom decreases significantly.

On the basis of the results of two-way analysis of variance and the Duncan test (**Figure 4**; **Table 3**), this study revealed that, in the seabuckthorn plantation, the stand age and relative height had significant effects on the photosynthetic parameters and canopy structure indicators. Combinations of uppercase and lowercase letters are used to present the different significance groupings among the combined treatment groups (4 stand ages × 3 relative heights). Uppercase letters indicate the comparison results along the stand age sequence, and lowercase letters indicate the comparison results along the relative height gradient. For example, “Aa”, “Ba”, and “Ca” indicate that the stand age groups are A, B,and C, respectively, and that the relative height group is “a” for all of them; these are significantly different from the other letter groups for stand age and from the other letter groups for relative height.

**Table 3:** Differences of each index under different stand ages and relative heights [The lowercase letters (a-e) are significance grouping markers obtained from multiple comparisons among the 12 treatment combinations (4 stand ages × 3 relative heights)].

Stand age	Relative height	PPFD	PAR	PALe	OmegaE	LAI	Gap fraction
3	0 0.5	126.97 ± 29.23e 141.21 ± 24.46e	77.50 ± 17.84e 86.19 ± 14.93e	1.64 ± 0.72c 1.10 ± 0.30c	0.94 ± 0.08a 0.92 ± 0.09a	1.75 ± 0.75c 1.21 ± 0.35c	0.11 ± 0.08c 0.16 ± 0.08c
	1	578.34b	352.99b	0.07c	0.99a	0.07c	0.92a
4	0 0.5	406.79 ± 126.70b 353.18 ± 79.08b	248.29 ± 77.33b 215.56 ± 48.27b	1.66 ± 0.61c 1.42 ± 0.33c	0.91 ± 0.08a 0.91 ± 0.07a	1.81 ± 0.65c 1.56 ± 0.33c	0.24 ± 0.13b 0.22 ± 0.09b
	1	626.71b	382.52b	0.12c	0.93a	0.13c	0.86a
8	0 0.5	217.19 ± 87.38d 331.51 ± 128.23c	132.56 ± 53.33d 202.34 ± 78.27c	3.49 ± 1.07b 3.10 ± 1.43b	0.95 ± 0.06a 0.92 ± 0.08a	3.70 ± 1.14b 3.35 ± 1.49b	0.11 ± 0.07c 0.13 ± 0.10c
	1	1148.08a	700.73a	0.05c	1.00a	0.05c	0.96a
11	0 0.5	112.16 ± 38.45e 147.09 ± 49.83d	68.46 ± 23.47e 89.77 ± 30.42d	4.46 ± 1.20a 3.97 ± 1.22a	0.82 ± 0.06b 0.81 ± 0.09b	5.43 ± 1.24a 4.92 ± 1.35a	0.04 ± 0.04d 0.05 ± 0.03d
	1	808.98a	493.76a	0.18c	0.99a	0.19c	0.85a

Relative height definition: 0 = bottom of the canopy; 0.5 = middle of the canopy; 1 = top of the canopy.

Relative height had a significant effect on OmegaE (*F* = 3.26, *p* = 0.040), and highly significant effects on all other indicators (e.g., for PPFD: *F* = 88.78, *p* < 0.001); the other indicators were also highly significant (for example, PPFD: *F* = 88.75, *p* < 0.001); however, the interaction effect between the two was highly significant only for PPFD (*F* = 10.15, *p* < 0.001) and PAR (*F* = 10.15, *p* < 0.001) (**Table 4**). Duncan *post hoc* tests further revealed that LAI (5.01, group A) and PALe (4.09, group A) reached the maximum when the stand age increased to the 11th year, but PPFD (151.54, group C) and PAR (92.49, group C) were significantly lower than those in the 4th year (PPFD: 384.03, group A; PAR: 234.39, group A). In the vertical distribution, the top canopy (relative height 1.0) had the highest PPFD (790.53, group A) and Gap Fraction (0.90, group A), but its LAI (0.11, group b) and PALe (0.11, group b) were the lowest; PAR increased significantly with increasing relativeheight, reaching its highest value at the top (relative height 1.0) and the lowest at the bottom, OmegaE showed little variation across different stand ages and relative heights, generally ranging between 0.9 and 1.0, but decreased slightly at the bottomof the 11−year−old stand (to approximately 0.82). The results, including specific statistical data and mean values, clearly describe the quantitative laws of the photosynthetic characteristics and canopy structure of seabuckthorn with increasing stand age and vertical height, which provides accurate data for understanding its ecological adaptability and formulating management strategies. By precisely quantifying the specific thresholds and interaction intensities in seabuckthorn plantations, management decisions for these plantations can be directly guided.

**Table 4:** Differences of each index under different stand ages and relative heights (ANOVA results).

Dependent variable	Stand age (F, p)	Relative height (F, p)	Interaction (F, p)
PPFD	105.26,<0.001	105.56,<0.001	88.75,<0.001
PAR	105.56,<0.001	88.75,<0.001	10.15,<0.001
PALe	102.41,<0.001	20.23,<0.001	1.26, 0.2762
OmegaE	18.42,<0.001	3.26,<0.05	0.44, 0.8488
LAI	133.92,<0.001	21.94,<0.001	1.69, 0.126
Gap Fraction	34.09,<0.001	149.96,<0.001	1.40, 0.217

**Figure 5:**
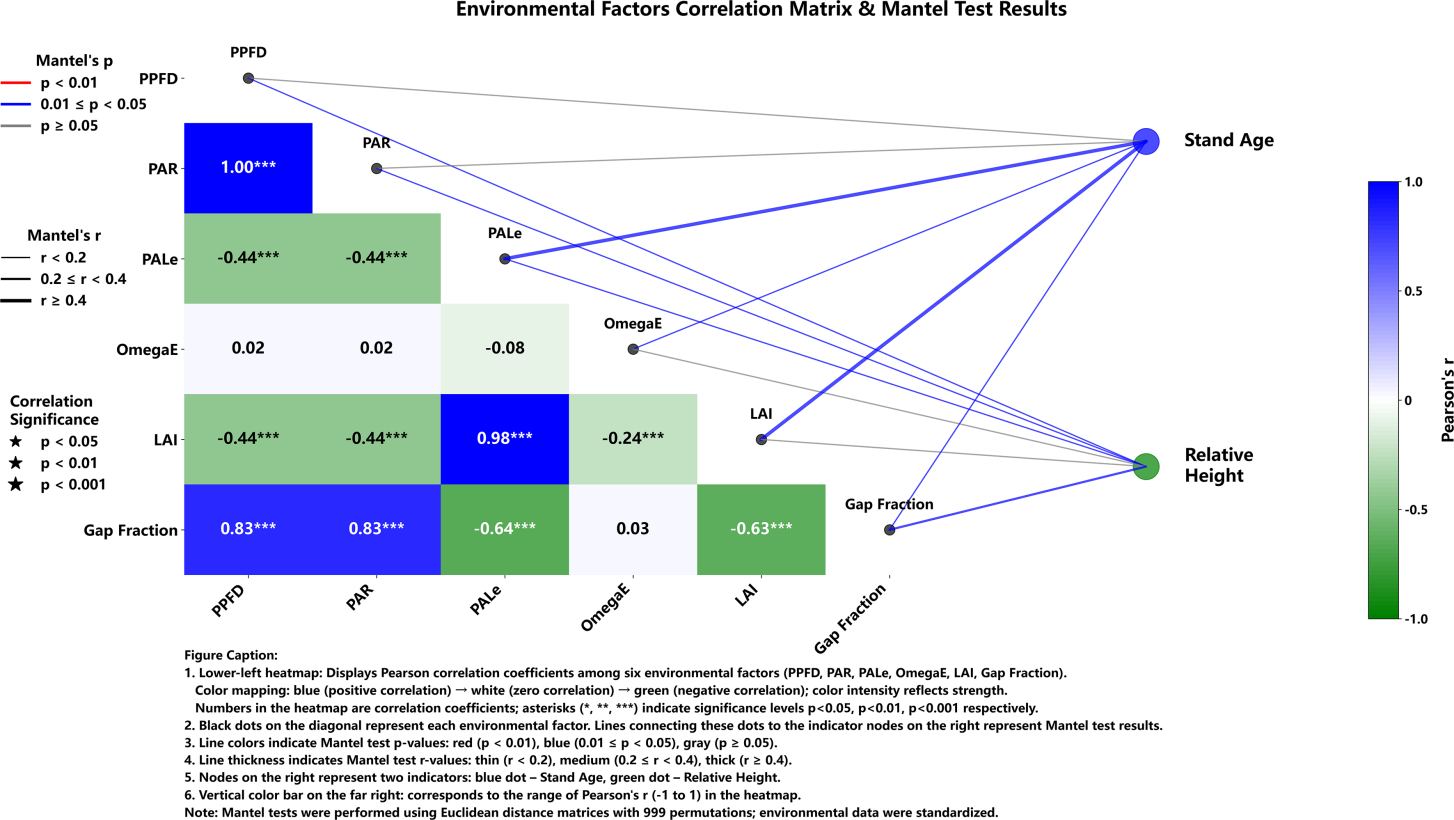
Mantel test analysis.

**Figure 6:**
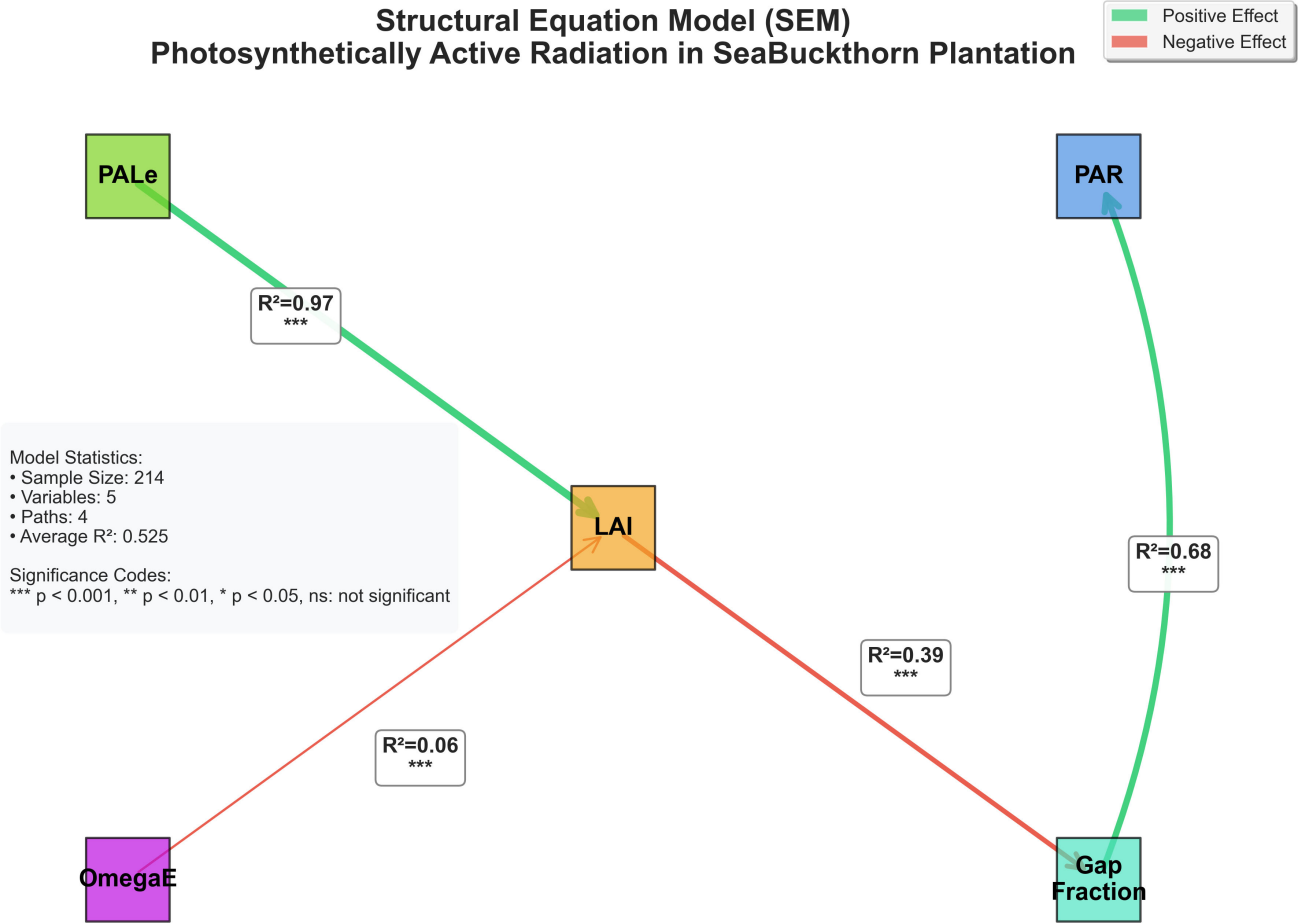
SEM analysis.

On the basis of the results of the Mantel test and SEM (**Figures 5**, **6**), this study further revealed the relationships among the environmental factors, spatiotemporal variables (stand age, relative height) and internalindicators of the seabuckthorn plantation. The Mantel test revealed that PALe and LAI were significantly positively correlated with stand age (Mantel *r* = 0.526, *p* = 0.010; Mantel *r* = 0.593, *p* = 0.010), and the Gap Fraction was also significantly positively correlated with relative height (Mantel *r* = 0.278, *p* = 0.010), indicating that the increase in stand age mainly drives the development of canopy structure, whereas the vertical position of the canopy dominates the light distribution pattern.

Since there is a clear linear relationship between PPFD and PAR (**Equation 7**), the PPFD index is not added to the analysis, and the linear relationship is as follows:

(7) PAR=(PPFD/4096)×2500

SEM path analysis further quantifies the key causal relationship (**Table 5**): canopy structure is the core of driving light resource distribution, and the Gap Fraction has a strong direct positive effect on PAR (*β* = 0.826, *R*^2^ = 0.682, *p* < 0.001); at the same time, PALe has almost complete explanatory power for the LAI (*β* = 0.983, *R*^2^ = 0.966, *p* < 0.001), whereas the LAI has a significant direct negative effect on the Gap Fraction (*β* = 0.628, *R*^2^ = 0.394, *p* < 0.001). In addition, although OmegaE had a significant negative effect on the LAI (*β* = −0.239, *p* < 0.001), its explanatory power was weak (*R*^2^ = 0.057). Taken together, these results confirm that in the seabuckthorn plantation ecosystem, canopy structure (especially the Gap Fraction and LAI) is a key mediator connecting stand age, vertical height and the light environment and constitutes the core path of “stand age/height → canopy structure → light resource distribution”.

**Table 5:** Path coefficient summary.

Path	Coefficient (*β*)	*R*2	Correlation (*r*)	*p*-value	Significance
Gap Fraction → PAR	0.826	0.682	0.826	0.0000	***
PALe → LAI	0.983	0.966	0.983	0.0000	***
OmegaE → LAI	-0.239	0.057	-0.239	0.0004	***
LAI → Gap Fraction	-0.628	0.394	-0.628	0.0000	***

"***" indicates the level of statistical significance, specifically meaning p < 0.001.

### 3.2 Regulation of light distribution by stand age, relative height and canopy structure

**Figure 7:**
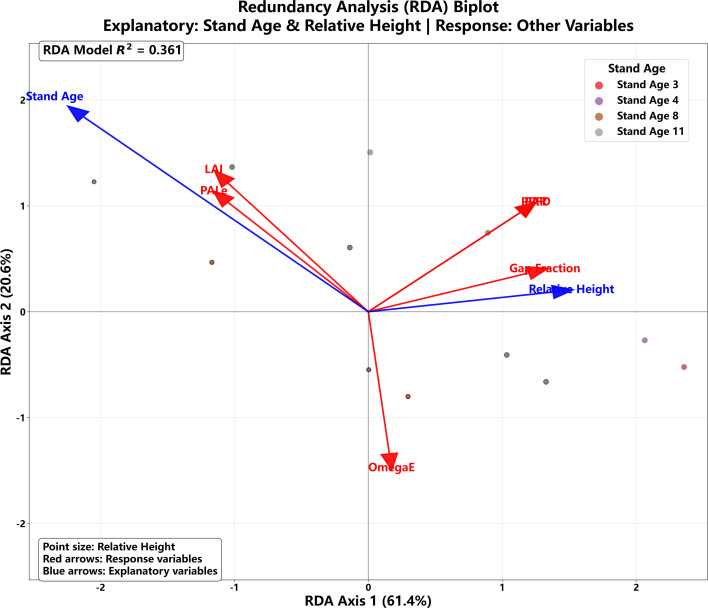
RDA analysis.

On the basis of the results of RDA (**Figure 7**), this study clarified the comprehensive influence patterns of stand age and relative height on multiple indicators of the seabuckthorn plantation ecosystem. The RDA model is significant as a whole, and the two explanatory variables together explain 36.07% of the total variance of the system (*R*^2^ = 0.3607). The explanatory power of stand age was significantly greater than that of relative height, with an independent contribution rate of 31.72%, whereas the relative height was only 4.27%.

In the RDA ranking space, the stand age vector (coordinates: [-0.8517, 0.7371]) and the relative height vector (coordinates: [0.5517, 0.0745]) presented significantly different directional characteristics, indicating that there were essential differences in the mechanism of their effects on the seabuckthorn plantation. The loading analysis of the response variables revealed that LAI (coordinates: [-0.4197, 0.4869]) and PALe (coordinates: [-0.4195, 0.4137]) were clustered in the RDA space and were close to the direction of the stand age vector, whereas PPFD (coordinates: [0.4549, 0.3754]) and PAR (coordinates: [0.4549, 0.3754]) were more consistent with the direction of the relative height vector.

The multiple regression *R*^2^ values of each response variable further confirmed this pattern. Stand age and relative height had the strongest explanatory power for LAI (*R*^2^ = 0.6407) and PALe (*R*^2^ = 0.5877), whereas the explanatory power for PPFD and PAR was relatively weak (*R*^2^ = 0.0700). These results systematically reveal the ecological law that stand age mainly drives the development of canopy structure (such as LAI and PALe), whereas relative height (vertical position) dominates thedistribution pattern of light resources (such as PPFD and PAR).

### 3.3 Establishment of a seabuckthorn classification model on the basis of stand age

**Figure 8:**
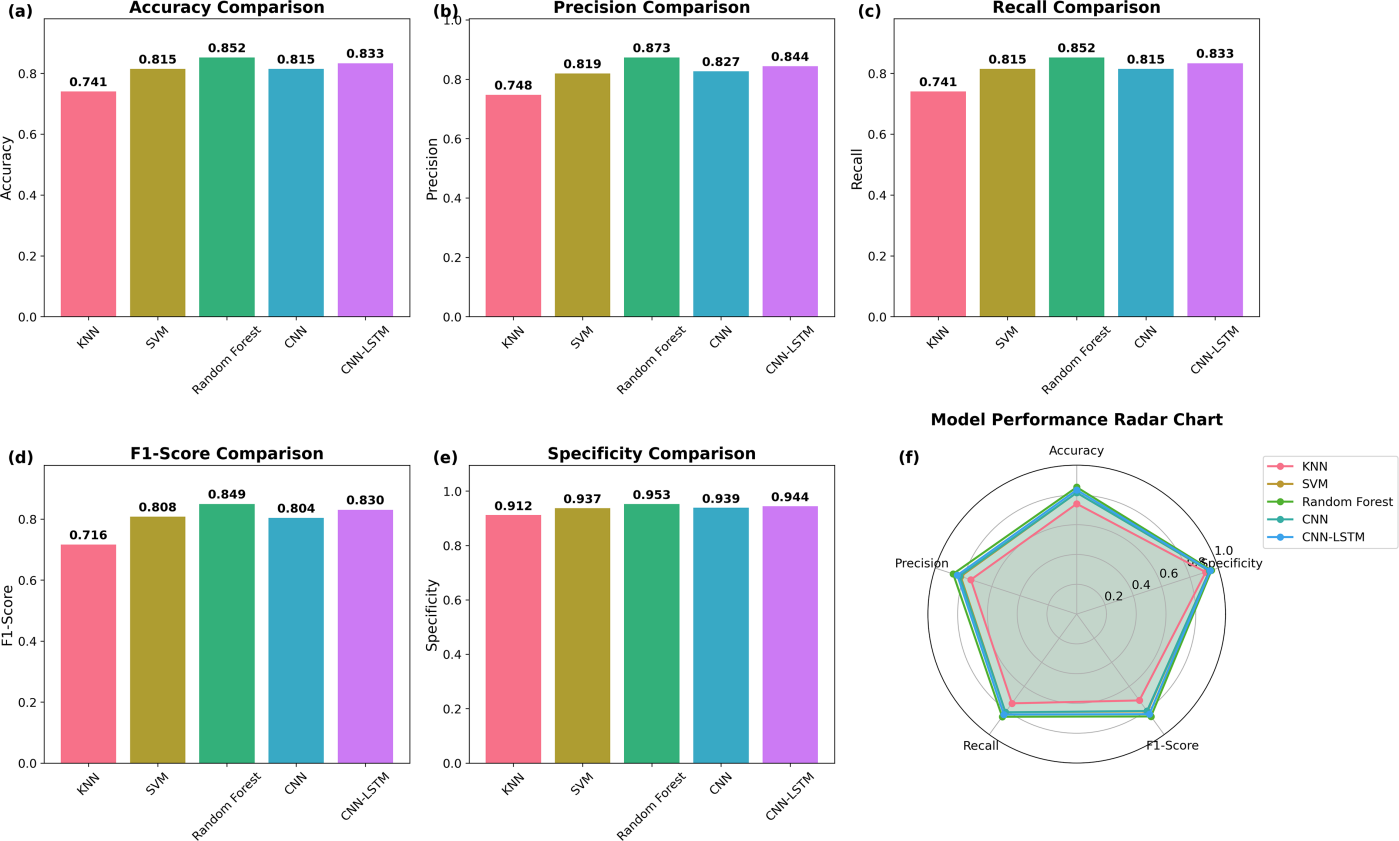
Comparison of the effects of the five models. **(a)** Statistics of the five models in terms of accuracy; **(b)** statistics of the five models in terms of precision; **(c)** statistics of the five models in terms of recall; **(d)** statistics of the five models in terms of F1-score; **(e)** statistics of the five models in terms of specificity; **(f)** comprehensive performance of the KNN, SVM, Random Forest, CNN, and CNN-LSTM models.

Based on a training set of 75% and a test set of 25% derived from 214 samples (which include stand age, relative height, and six ecological indicators), the machine learning modeling results indicate that the random forest model performed best in the seabuckthorn plantation classification task (**Figure 8**; **Table 6**). For 54 test samples, the random forest achieved an accuracy rate of 85.19%. The accuracy rate, recall rate and F1 score were 87.29%, 85.19% and 84.94%, respectively. The specificity was as high as 95.25%, and the comprehensive performance was the best. The CNN-LSTM model ranks second with an accuracy of 83.33%, and its accuracy, recall, and F1 score are 84.38%, 83.33%, and 83.04%, respectively. Among the traditional machine learning methods, the SVM yields an accuracy of 81.48%, whereas the KNN is relatively low, at 74.07%. For the deep learning method, the CNN model achieves an accuracy of 81.48%, which is comparable to that of the SVM. The performance of each model is ranked as follows: random forest *>* CNN-LSTM *>* SVM ≈ CNN *>* KNN. This result confirms the advantages of the ensemble learning method based on the tree model in the classification of seabuckthorn plantation ecological data and provides a reliable technical approach for using ecological indicators to invert seabuckthorn plantation age.

**Table 6:** Model effect parameters.

Model	Accuracy	Precision	Recall	F1-Score	Specificity
KNN	0.7407	0.7477	0.7407	0.7164	0.9117
SVM	0.8148	0.8194	0.8148	0.8079	0.9373
Random Forest	0.8519	0.8729	0.8519	0.8494	0.9525
CNN	0.8148	0.8269	0.8148	0.8039	0.9385
CNN-LSTM	0.8333	0.8438	0.8333	0.8304	0.9439

## 4 Discussion

### 4.1 Age-driven canopy structure and light environment evolution in economic forests

Through systematic quantitative analysis, this study revealed the dynamic evolution of the canopy structure and light environment of seabuckthorn plantation with stand age and provided important empirical data for forest ecology research. From 3 years of age to 11 years of age, LAI of seabuckthorn plantation increased significantly, increasing by nearly 2.5 times, from approximately 1.2 to approximately 4.3. This significant increase in the LAI reflects the rapid expansion of the number and area of leaves during the growth of seabuckthorn plantation, which in turn leads to significant changes in canopy structure. With increasing LAI, the canopy gap rate decreased significantly, from approximately 35% to approximately 10%. A decrease in the canopy gap rate means that the canopy’s ability to intercept light is enhanced and that the light environment in the forest has changed significantly. This change in canopy structure directly led to a significant change in the light environment in the forest, especially a significant decrease in PAR in the middle and lower layers. This change had a profound effect on the growth and community structure of understory plants from 70% to 20% full light.

This ecological process of ‘growth-closing-shading’is a universal law of forest development. This study provides a clear demonstration of seabuckthorn plantation through continuous stand age sequence data (Matsuo et al., 2021). This process not only reflects the changes in the internal structure of the seabuckthorn forest during the growth process but also reveals the driving effect of stand age on the understory environment and community succession (Zhang and Chen, 2007; Atkins et al., 2018). Resource competition caused by stand age, especially the redistribution of light resources within the canopy, is a key driving force for understory environmental change and community succession (Shan et al., 2025). The process of “growth canopy shading” of seabuckthorn economic forests with increasing stand age is the key stage of the gradual transition from vegetative growth to reproductive fruiting (Shi et al., 2025). The change in canopy structure is directly related to the formation of fruit yield and quality and the redistribution of light resources in the canopy, which affects the spatial distribution and photosynthetic efficiency of fruiting branches (Zhang et al., 2022). With the decrease in light in the forest, the fruit part may gradually move, which affects the picking efficiency and labor cost (Anthony and Minas, 2025). The reduction in understory light also restricts the application of understory economic plants or intercropping patterns, which suggests that in the management of economic plantations, it is necessary to optimize the distribution of canopy light and coordinate the relationship between growth and fruiting through reasonable shaping and pruning, density regulation and other measures (Ligot et al., 2014). The results of this study are consistent with the law that yield formation in most economic forest tree species (such as trees and Camellia oleifera) is regulated by light with canopy closure, which further confirms the importance of maintaining a good light environment through structural management for sustainably high yields of economic forests in the process of increasing stand age (Plaga et al., 2024; Liao et al., 2025; Shen et al., 2025). The economic benefits of seabuckthorn plantations depend not only on site conditions and variety characteristics but also on canopy structure management. In the cultivation of seabuckthorn economic plantations, appropriate management measures should be formulated according to the dynamic laws of the canopy and light environment, such as timely thinning and reasonable pruning, to maintain canopy permeability and promote effective fruiting to improve the overall economic benefits and the comprehensive utilization value of forestland (Forrester et al., 2013).

### 4.2 Mechanism of canopy structure regulation of the understory light environment

In this study, the complex relationship between the canopy structure and the understory light environment of a seabuckthorn plantation was analyzed via SEM, and the key role of the unit gap area in controlling understory PAR transmission was revealed. The goodness of fit of the model is good, *χ*^2^*/df* is less than 3, the CFI is greater than 0.95, and the RMSEA is less than 0.08. These indicators indicate that the model has a very good fitting effect on the data and can reliably reflect the relationships between variables. The results of path analysis revealed that although LAI was the most significant parameter with age, its standardized path coefficient *β* value was -0.85, and the *p*-value was less than 0.001, indicating very high significance, the effect of the LAI on the PAR was achieved mainly by negatively changing the canopy gap rate. The standardized path coefficient of the canopy gap rate was -0.78, and the *p*-value was less than 0.001, indicating that it had a strong indirect effect on the PAR. Specifically, the indirect effect of LAI on PAR accounted for approximately 70% of the total effect, while its direct effect was relatively small. The standardized path coefficient was only 0.15, and the *p*-value was greater than 0.05, which did not reach a significant level.

This result profoundly suggests that in forest management, direct assessment and regulation of canopy openness (such as Gap Fraction) may be more direct and effective than relying solely on the LAI for predicting and optimizing the understory light environment (Sani-Mohammed et al., 2024). This not only provides a new perspective for forest management but also provides a more targeted strategy for optimizing the understory light environment. This study also revealed that there was a close correlation between PALe, which contains nonleaf organ information, and the LAI, and the *R*^2^ value was 0.89, indicating that there was a strong correlation between the two. This result shows that PALe, a three-dimensional structure index, can more fully reflect the true structural complexity of canopy interception and scattering of light in canopies with complex branching structures, such as seabuckthorn (Jonckheere et al., 2004). In the management of forest ecosystems, understanding the effects of canopy structure on the light environment is important (Alveshere et al., 2024). Chenu Karine et al. proposed a method to estimate light interception by using three-dimensional virtual plant modeling and directional light characteristics, which is suitable for highly uneven light environments. This method can be used to calculate the light interception efficiency of different genotypes of plants effectively in natural and artificial environments and is highly important for studying the genotype-environment interaction between plant structure and light interception efficiency (Chenu et al., 2008). Tang et al. analyzed the influence of the branch angle of loquat trees on light interception through a three-dimensional canopy model and reported that a reasonable branch angle could be used to optimize light distribution and improve light interception efficiency, providing a new digital and visual tree structure design method for fruit farmers (Tang et al., 2019). The above studies verified that the introduction of the PALe three-dimensional structural index of the seabuckthorn canopy not only enriches the quantitative methods for determining canopy structure but also provides a more effective tool for forest management. By directly regulating the canopy gap rate, the understory light environment can be predicted and optimized more accurately to better meet the management objectives of seabuckthorn plantation ecosystems, such as improving biodiversity, promoting the growth of understory vegetation and maintaining ecosystem stability.

### 4.3 Inspiration for the management of seabuckthorn plantation

Through in-depth analysis of the structural characteristics and light environment of seabuckthorn plantation, this study reveals differentiated management strategies for seabuckthorn plantation under different management objectives and provides clear guidance for the sustainable development of seabuckthorn plantation.

For seabuckthorn plantation with the main goals of ecological protection, such as soil and water conservation and windbreak and sand fixation, this study suggests maintaining or cultivating stand structures with relatively high stand ages (such as more than 10 years) and high LAI (such as more than 4.0). This structure can form a closed canopy and maximize the canopy’s effect on the surface (Mwangi et al., 2025). In arid and semiarid regions, this shading effect is crucial for ecological restoration (Patil et al., 2024). This model can effectively inhibit the competition of understory weeds, reduce the evaporation of soil moisture, and promote the accumulation of litter (Gomez-Cifuentes et al., 2020). The accumulation of litter can not only increase the content of soil organic matter and improve the soil structure but also further improve the water retention capacity of the soil, form a virtuous cycle, and promote the stable growth of seabuckthorn plantation and the restoration of ecosystems (Castagnolli et al., 2023). For seabuckthorn economic plantations, which have the main goals of fruit harvesting and economic benefits, this study proposes a more refined management strategy. Studies have shown that when the stand age of a seabuckthorn plantation reaches a certain stage (such as 8 years later) or the canopy PAR decreases to a critical level (such as less than 30% full light), timely intervention measures need to be taken. The canopy gap rate can be moderately increased to 15%-25% through reasonable shaping and pruning, such as open-center pruning, evisceration of boreal branches, or low-intensity target tree thinning (Lima and Moura, 2006). This moderate canopy gap rate can effectively improve the light conditions in the canopy, especially the light conditions in the fruiting branch area (Tang et al., 2019). Adequate light has been shown to significantly promote the flower bud differentiation of seabuckthorn and improve the fruit setting rate, thereby increasing fruit yield (Du et al., 2023). The improvement of light conditions can also significantly improve fruit quality, such as increasing the vitamin C content and oil content in fruit (Jaśniewska and Diowksz, 2021). The improvement of these qualities can not only increase the market value of the fruit but also further improve the economic benefits of the seabuckthorn economic plantation (Marappan et al., 2025). This study suggests that the precise regulation of the light environment via canopy structure monitoring is a key technical way to achieve synergistic improvements in the ecological and economic benefits of seabuckthorn plantation. Through this precise regulation, the economic benefits of seabuckthorn plantation can be maximized while meeting the needs of ecological protection, and a win-win situation in terms of ecology and the economy can be achieved.

## 5 Conclusion

The results of this study clearly revealed the dynamic evolution of the canopy structure and light environment of seabuckthorn, an ecologically important economic plantation, with increasing stand age. From 3 years of age to 11 years of age, LAI of seabuckthorn plantation significantly increased by approximately 2.5 times (from approximately 1.2 to approximately 4.3), whereas the canopy gap rate decreased from approximately 35% to approximately 10%, resulting in a sharp decrease in PAR of the middle and lower canopies from approximately 70% full light to approximately 20%. This process of “growth-closing-shading” marks the key stage of the seabuckthorn plantation from vegetative growth to reproductive fruiting. The canopy structure (represented by the LAI and Gap Fraction) is a key intermediary between stand age and the light environment in the plantation, which directly regulates the distribution pattern of light resources in the resulting area. The random forest model can classify stand age according to canopy structure parameters with an accuracy of 85.19%, which provides a reliable technique for inverting the plantation development stage and guiding precise management by monitoring the canopy state. These findings will provide important support and impetus for the future management and development of seabuckthorn plantations.

## Data Availability

The original contributions presented in the study are included in the article/supplementary material. Further inquiries can be directed to the corresponding authors.
